# THE HOSPITAL ENVIRONMENT’S INFLUENCE ON THE INCLUSION OF CARE PARTNERS OF HOSPITALIZED PLWD

**DOI:** 10.1093/geroni/igae098.2751

**Published:** 2024-12-31

**Authors:** Catherine Still, Sydney Hoel, Andrea Strayer, Nicole Werner, Beth Fields

**Affiliations:** University of Wisconsin Madison, Madison, Wisconsin, United States; Indiana University, Bloomington, Bloomington, Indiana, United States; University of Iowa, Iowa City, Iowa, United States; Indiana University, Bloomington, Bloomington, Indiana, United States; University of Wisconsin Madison, Madison, Wisconsin, United States

## Abstract

Federal initiatives like the National Strategy to Support Family Caregivers advocate for the inclusion of care partners into their family member or friends care teams. Yet, care partners of hospitalized people living with dementia (PLWD) often report feeling left out of the hospital care. This feeling of exclusion may be related to the hospital’s physical environment. Prior research has explored dementia care partners’ perceptions of the hospital space, but there is little research examining how the hospital environment can facilitate or hinder care partner inclusion. Therefore, this study sought to explore how the physical environment of the hospital influences care partner inclusion practices. Our team used a descriptive qualitative approach and collected data from two sources: direct observations (27 hours) in a large academic hospital and interviews with care partners (15) and clinicians/administrators (23) representing hospital experiences across the country. Observational data were analyzed using a framework analysis, and interview data were analyzed using a team-based inductive content analysis. Observational data revealed an underutilization of environmental resources, including communication tools and family-dedicated spaces. Interview data revealed three aspects of the physical environment that influence care partner inclusion: space for both the PLWD and their care partner, room personalization, and parking. Health systems should ensure adequate space for care partners in the hospital, allow care partners to personalize patient rooms, and designate dementia-friendly parking spaces. Modification of the physical environment may promote the inclusion of care partners into hospital care, which could potentially improve the health outcomes for hospitalized PLWD.

